# Feline panleukopenia virus in cerebral neurons of young and adult cats

**DOI:** 10.1186/s12917-016-0657-0

**Published:** 2016-02-19

**Authors:** Mutien Garigliany, Gautier Gilliaux, Sandra Jolly, Tomas Casanova, Calixte Bayrou, Kris Gommeren, Thomas Fett, Axel Mauroy, Etienne Lévy, Dominique Cassart, Dominique Peeters, Luc Poncelet, Daniel Desmecht

**Affiliations:** Department of Morphology and Pathology, University of Liège, Liège, Belgium; Department of Clinical Sciences, University of Liège, Liège, Belgium; Department of Infectious and Parasitic Diseases, Centre for Fundamental and Applied Research for Animals & Health, Faculty of Veterinary Medicine, University of Liège, Liège, Belgium; Laboratory of Anatomy, Biomechanics and Organogenesis, Faculty of Medicine, Free University of Brussels, Brussels, Belgium

**Keywords:** Feline panleukopenia virus, Cat, Neurons, Neurological disorders

## Abstract

**Background:**

Perinatal infections with feline panleukopenia virus (FPV) have long been known to be associated with cerebellar hypoplasia in kittens due to productive infection of dividing neuroblasts. FPV, like other parvoviruses, requires dividing cells to replicate which explains the usual tropism of the virus for the digestive tract, lymphoid tissues and bone marrow in older animals.

**Results:**

In this study, the necropsy and histopathological analyses of a series of 28 cats which died from parvovirus infection in 2013 were performed. Infections were confirmed by real time PCR and immunohistochemistry in several organs. Strikingly, while none of these cats showed cerebellar atrophy or cerebellar positive immunostaining, some of them, including one adult, showed a bright positive immunostaining for viral antigens in cerebral neurons (diencephalon). Furthermore, infected neurons were negative by immunostaining for p27^Kip1^, a cell cycle regulatory protein, while neighboring, uninfected, neurons were positive, suggesting a possible re-entry of infected neurons into the mitotic cycle. Next-Generation Sequencing and PCR analyses showed that the virus infecting cat brains was FPV and presented a unique substitution in NS1 protein sequence. Given the role played by this protein in the control of cell cycle and apoptosis in other parvoviral species, it is tempting to hypothesize that a cause-to-effect between this NS1 mutation and the capacity of this FPV strain to infect neurons in adult cats might exist.

**Conclusions:**

This study provides the first evidence of infection of cerebral neurons by feline panleukopenia virus in cats, including an adult. A possible re-entry into the cell cycle by infected neurons has been observed. A mutation in the NS1 protein sequence of the FPV strain involved could be related to its unusual cellular tropism. Further research is needed to clarify this point.

## Background

Feline panleukopenia virus (FPV) and canine parvovirus (CPV) both belong to the *Protoparvovirus* genus within the *Parvovirinae* subfamily of the *Parvoviridae* family of single-stranded DNA viruses [1]. CPV-1 and 2 infect *Canidae* and CPV-2 emerged as a new host range variant in the mid-1970s (CPV-2) and spread worldwide in the canine population in 1978 [2]. Then, antigenic variants CPV-2a, b, c have gained infectivity for other species such as cats. FPV and FPV-like strains (such as mink enteritis virus, MEV) are unable to infect *Canidae* [1, 3]. Although most FPV and CPV strains have been isolated from cats and dogs, a broad range of alternative hosts have been identified within the *Carnivora* order [1].

Parvovirus genome replication takes place in the nucleus and requires cells in S phase, since it relies on host cell machinery for the formation of double-stranded replication intermediates [4, 5]. This requirement limits the tropism of FPV and CPV to highly dividing cells such as those found in the intestine, bone marrow or lymphoid tissues. In kittens during the perinatal period, the infection of neuroblasts of the external granular layer is thought to be responsible for the cerebellar hypoplasia typically associated with such infections. However, viral proteins are expressed in some Purkinje cells despite the fact that these neurons are post-mitotic at this development stage [6, 7]. Nervous tissue infection by FPV has never been described in adult cats, although positive CPV immunostaining of feline cerebral neurons has been reported [8], which raises questions about the possible re-entry of some neurons into the S phase of the cell cycle, making them susceptible to infection.

In the present study, we show strong evidence of infection of cerebral neurons by FPV in young and adult cats, some of which with a history of neurological signs, associated with a unique mutation in the NS1 (nonstructural protein 1) amino acid sequence. Besides, one affected cat showed a co-infection by feline bocavirus type 1, which is the first evidence of nervous system infection by a bocavirus.

## Results

Twenty eight parvovirus-positive cats, aged from 6 weeks to 5 years (mean: 12.5 months +/− 17.5 months; Table 1) were investigated in this study. Real time PCR revealed the presence of parvovirus DNA in most organs tested, with the highest concentrations in the spleen, small intestine and mesenteric lymph node (mean C_T_ (Cycle Threshold) for these three organs: 19,3 +/− 2,9). Interestingly, brain tissues were positive for most cats, with relatively low C_T_ values (20–25) in several of them (cats No 5, 9, 10, 11, 14, 15 and 16). Especially, the differences in C_T_ value between brain tissues and the ileum (small intestine) were highly variable, ranging from 2.2 to 14.9. This difference was the lowest (<6) in cats No 5, 10, 11, 14 and 15. Several of these cats were reported by referring veterinarians to have shown neurological disorders before death, mostly ataxia and/or dysphagia.

**Table 1:** Details of the 28 FPV-positive cats included in the study

Cat number	Sex	Breed	Age
1	F	Persian	22 weeks
2	M	European shorthair	16 weeks
3	M	European shorthair	5 years
4	F	Maine Coon	4 years
**5**	**M**	**Maine Coon**	**6 weeks**
6	F	European shorthair	22 weeks
7	F	European shorthair	2.5 years
8	M	European shorthair	14 weeks
9	M	European shorthair	10 weeks
**10**	**F**	**European shorthair**	**10 weeks**
**11**	**M**	**European shorthair**	**4.5 years**
12	F	European shorthair	24 weeks
13	F	Siamese cross	20 weeks
14	M	European shorthair	14 weeks
**15**	**F**	**European shorthair**	**12 weeks**
16	M	European shorthair	11 weeks
17	M	European shorthair	5 months
18	M	European shorthair	1.5 years
19	F	European shorthair	2 years
20	F	European shorthair	4 years
21	F	European shorthair	14 weeks
22	M	European shorthair	1.5 years
23	F	European shorthair	24 weeks
24	F	European shorthair	14 weeks
25	F	Siamese	10 weeks
26	F	European shorthair	8 weeks
27	F	European shorthair	8 weeks
28	M	European shorthair	1 year

The four cats with positive immunostaining for FPV antigens in cerebral neurons are bolded

Affected cats were characterized on gross examination by mild to severe fibrinous to fibrinomucoid enteritis with thickened mucosa and highlighted Peyer’s patches and mesenteric lymphadenomegaly.

**Fig. 1:**
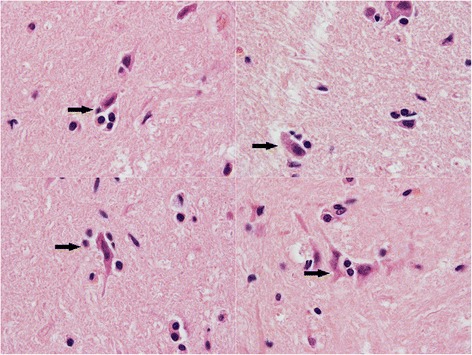
Histopathological features of brain sections (diencephalic region) from a 12-week-old parvovirus-infected cat presented in this study (original magnification x400). Satellitosis around neurons with condensed chromatine (neuronophagia) is observed

The histopathological analyses showed classical lymphocyte necrosis and depletion in lymphoid organs (Peyer’s patches and mesenteric lymph nodes) and severe intestinal villous blunting. Intestinal crypt cells were necrotic. Intestinal villi were depleted of their enterocytes and covered with a thick fibrinonecrotic exudate. Focal neuronal satellitosis and neuronophagic pictures (Fig. 1) were observed in the brain of cats No 5, 10, 11 and 15 (Table 1). None of the cats, even the youngest, showed any evidence of cerebellar atrophy.

**Fig. 2:**
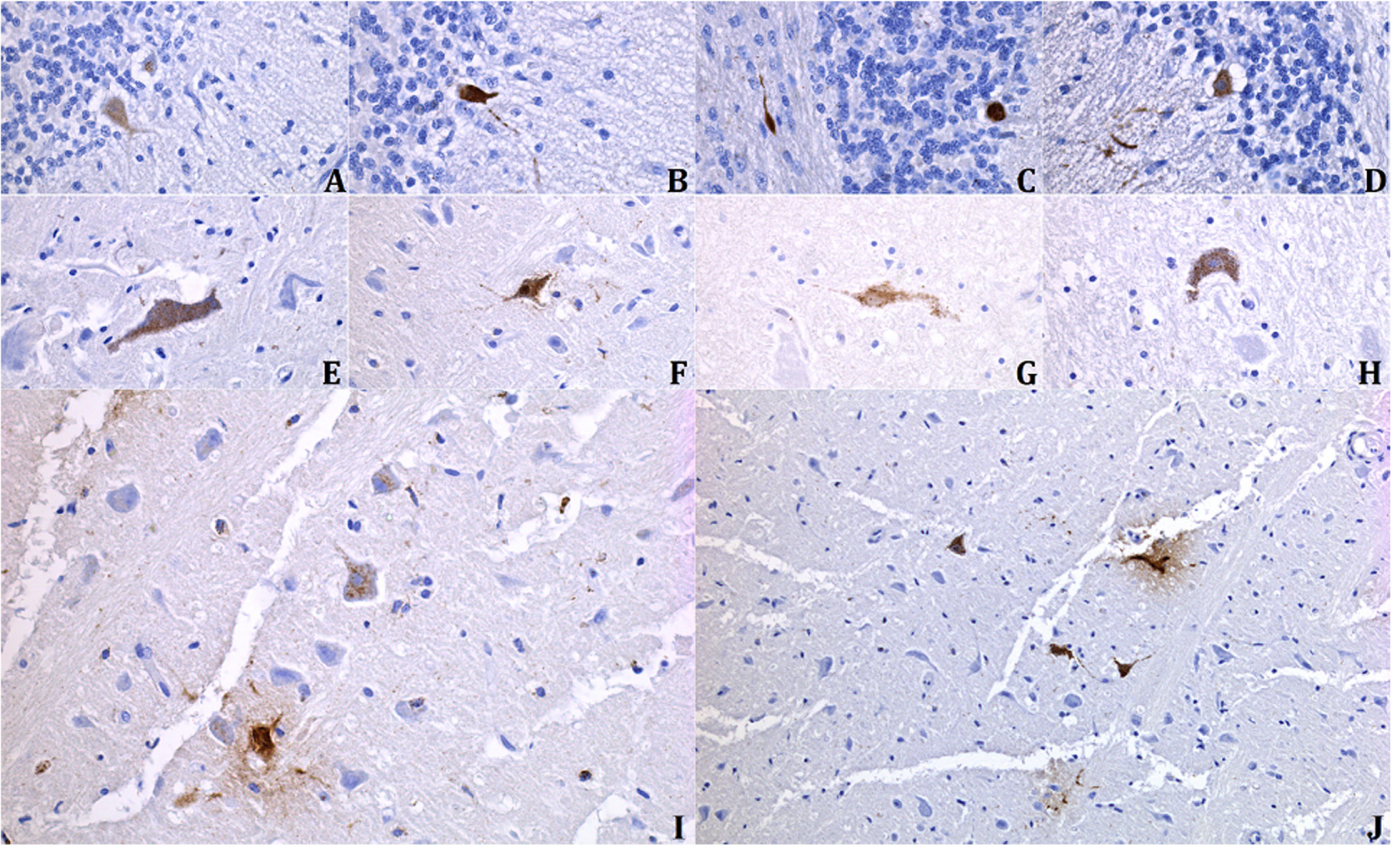
Immunohistochemical staining for FPV antigens in parvovirus-positive cat brains. **a** to **d** Intense cytoplasmic or nuclear immunostaining of Purkinje cells from an infected kitten cerebellum used as a positive control (original magnification x400). **e** to **j** Bright staining of neuronal bodies and processes and (**i**) several microglial cells in the diencephalon (interthalamic adhesion) from a 12-week-old cat presented in this study (original magnification x400; (**j**) original magnification x200)

**Fig. 3:**
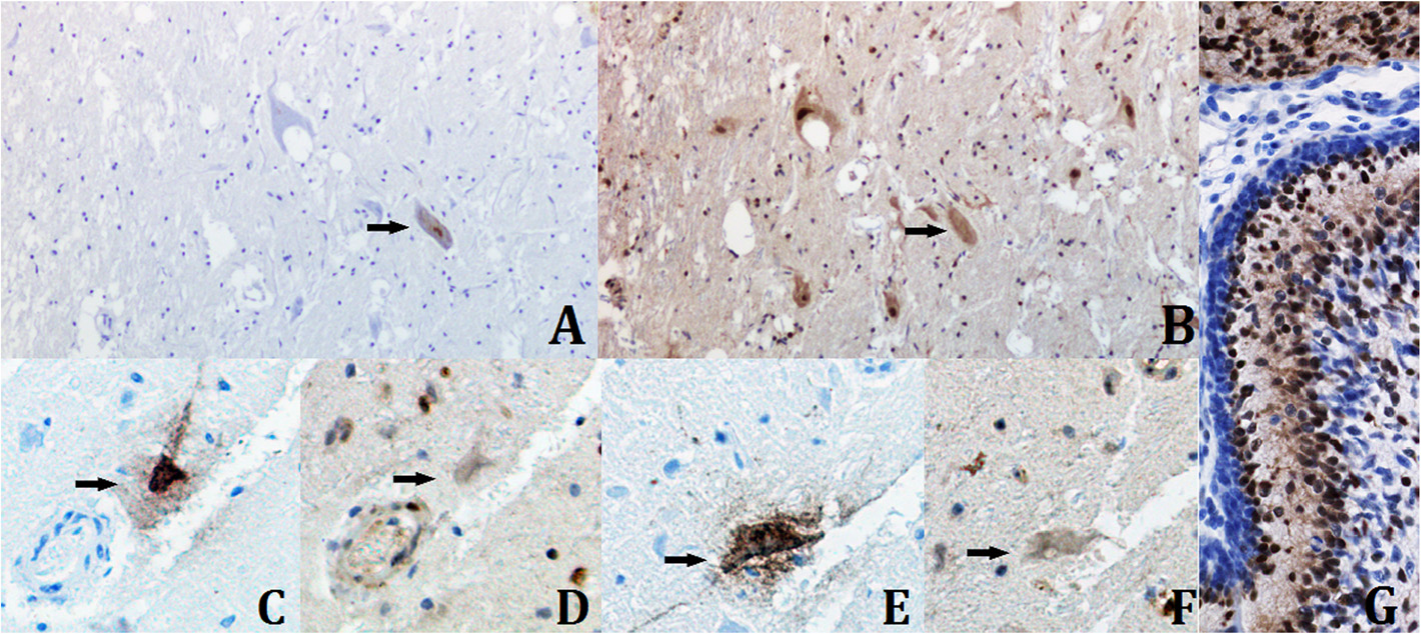
Immunohistochemical staining for FPV and p27^Kip1^ antigens. Immunostaining for FPV (**a**, **c**, **e**) and p27^Kip1^ (**b**, **d**, **f**) antigens on adjacent sections showing the absence of nuclear staining for p27^Kip1^ in FPV-infected neurons (**c**-**f**), while uninfected neurons still express nuclear p27 (**a**, **b**) (original magnification x100 (**a**, **b**) or x400 (**c** to **f**)). **g** Immunostaining for p27^Kip1^ antigen of cerebellar cortex from a feline fetus (estimated gestational age: 54 days) used as a positive control (original magnification x400)

Immunohistochemical (IHC) staining of the different sampled organs revealed the presence of parvoviral antigens in most locations. In particular, a bright staining was observed in cells from ileal crypts and follicular dendritic cells in the spleen and mesenteric lymph nodes. Staining was negative in the cerebellum but 10 cats (No 5, 10, 11, 12, 14, 15, 16, 22, 23, 26; Table 1) showed strong positivity for parvoviral antigens in other brain regions, especially the interthalamic adhesion of the diencephalon (Fig. 2). Glial cells (mostly microglial cells) but also neurons (in four cats, No 5, 10, 11 and 15) showed bright cytoplasmic staining. Infection was associated in some but not all infected neurons by signs of neuronal degeneration and glial reaction (Fig. 1). The four cats bearing infected neurons were 6 weeks, 10 weeks, 12 weeks and 4.5 years-old (cats No 5, 10, 11 and 15, Table 1). Further immunostaining of the brain from cat number 15 for p27^Kip1^ antigen revealed an absence of nuclear p27^Kip1^ expression in FPV-infected neurons, while it was clearly expressed in the nucleus of uninfected neurons (Fig. 3). A relatively strong background reaction could not be avoided with the antibody dilution (1/50) necessary to obtain a frank nuclear staining in p27^Kip1^ positive cells (Fig. 3).

**Fig. 4:**
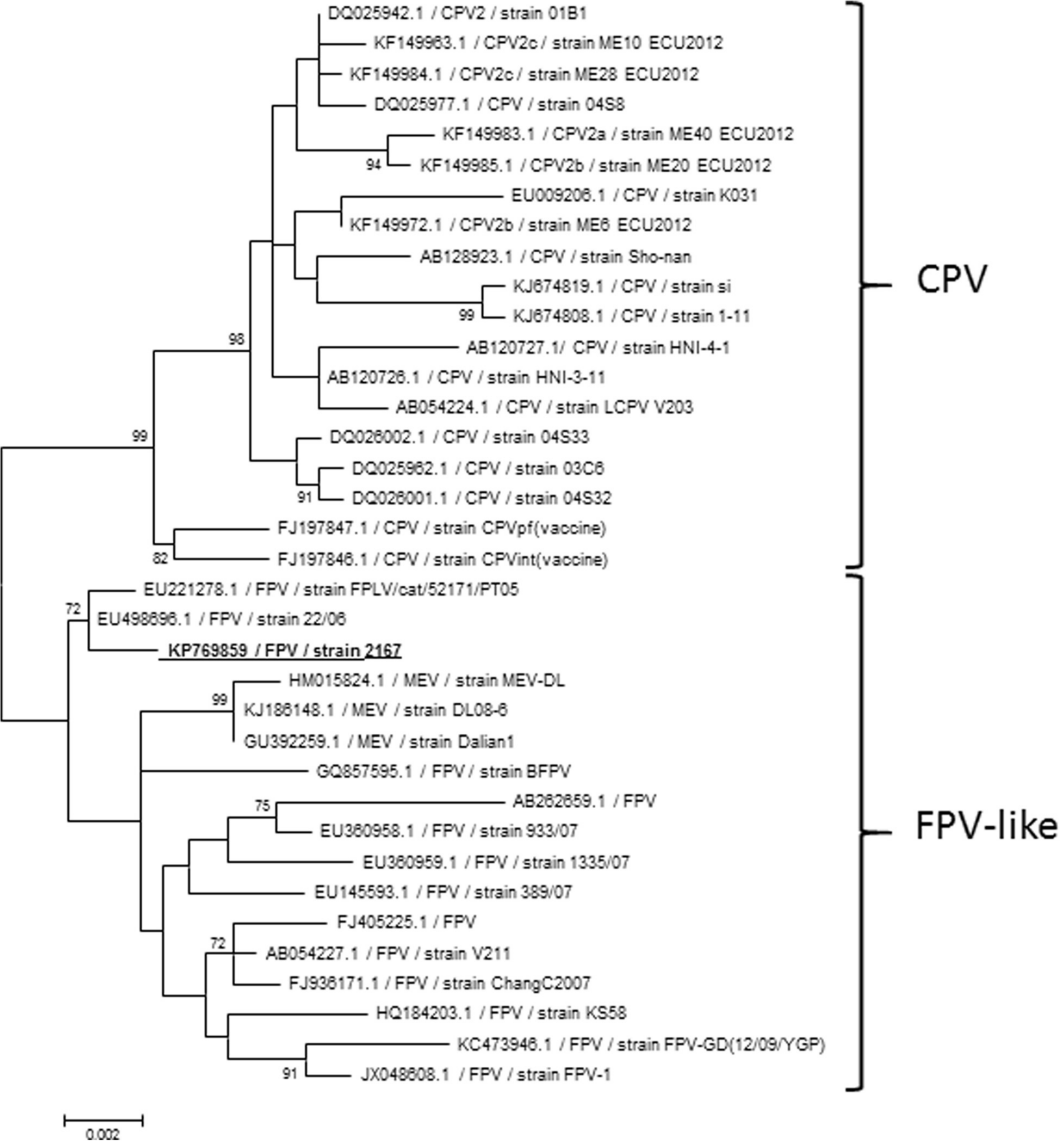
Maximum Likelihood phylogenetic analysis of the KP769859 FPV strain based on VP2 nucleotide coding sequence. Representative strains of canine parvovirus (CPV), feline panleukopenia virus (FPV) and mink enteritis virus (MEV) are included. Statistical support of 1000 parallel Maximum Likelihood bootstrap replicates (≥70 %) is indicated at the nodes. Taxon information includes GenBank accession number, name/antigenic variant and strain. The feline parvovirus strain generated in this study is bolded and underlined. The scale bar represents nucleotide substitutions per site

**Fig. 5:**
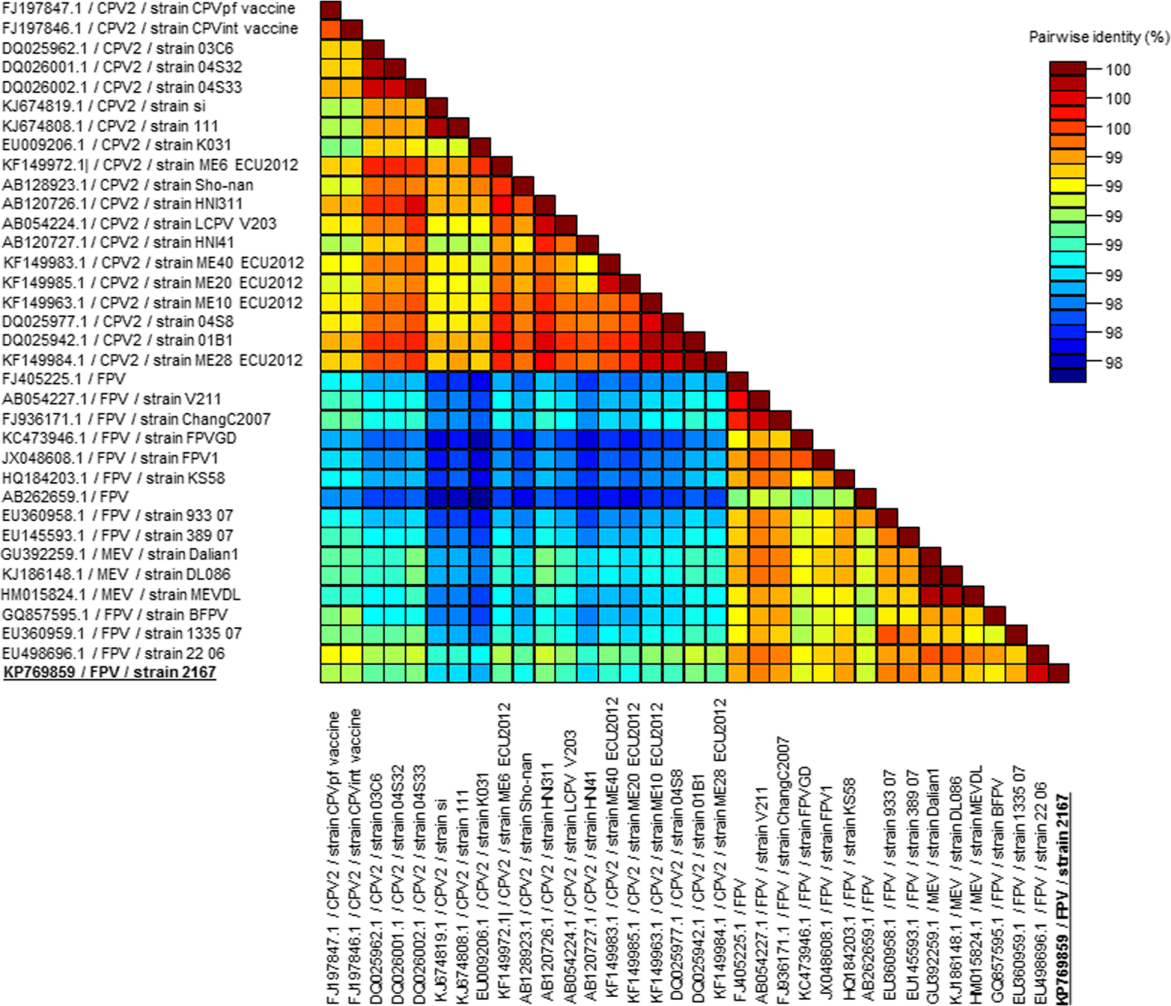
Pairwise identity matrix based on the viral VP2 gene sequence. A color-coded pairwise identity matrix based on the VP2 nucleotide coding sequence from representative strains of canine parvovirus (CPV), feline panleukopenia virus (FPV) and mink enteritis virus (MEV) reveals the KP769859 strain generated in this study belongs to the FPV group. The KP769859 feline parvovirus strain is bolded and underlined. A color key indicates the correspondence between pairwise identities and the colors displayed in the matrix

The full genome of the parvovirus present in the brain of the cat with the strongest IHC staining (cat No 5) was obtained using next-generation sequencing (GenBank accession number: KP769859). Sequence analysis allowed its classification as a feline panleukopenia virus, which was confirmed by a phylogenetic analysis (Fig. 4) and a sequence identity matrix (Fig. 5) based on the VP2 nucleotide coding sequence. A unique L → S substitution was observed at position 582 in the amino acid sequence of the NS1 protein (Table 2), resulting from a T → C nucleotide substitution at position 1745 of the NS1 coding sequence. Further sequence comparisons confirmed that this substitution had never been observed in FPV and CPV sequences available in GenBank to date. Sequences of VP1 and VP2 proteins were typical of FPV (Table 2).

**Table 2:** Parvovirus sequences from tissues of cats investigated (cats No 5, 10, 11, 15, 14, 18 and 23) in comparison with reference feline panleukopenia and canine parvovirus strains

Cat No or virus type/accession No	Origin	Amino acid position				
		VP2 aa 96	VP2 aa 106	VP2 aa 578	NS1 aa 248	NS1 aa 582
Cat No 5/KP769859	Cat - brain	K	V	A	T	S
Cat No 10	Cat - brain	K	V	A	T	S
Cat No 11	Cat - brain	K	V	A	T	S
Cat No 15	Cat - brain	K	V	A	ND	S
FPV/EU221279	Cat - fecal sample	K	V	A	ND	ND
FPV/BAA19024	Cat - ND	ND	ND	ND	T	L
CPV-2/FJ197847	Dog - fecal sample	N	A	G	ND	ND
CPV-2/NC_001539	Dog - cell culture	N	A	G	I	L
Cat No 14	Cat- ileum	ND	ND	ND	ND	L
Cat No 18	Cat- ileum	ND	ND	ND	ND	L
Cat No 23	Cat- ileum	ND	ND	ND	ND	L

ND: not determined

A PCR targeting the region of interest of NS1 coding sequence confirmed the unique substitution in the KP769859 strain. The same PCR was applied to DNA extracted from the other three cats (No 10, 11, 15) with neuronal IHC staining and revealed the presence of the same substitution (Table 2). Subsequent next-generation sequencing of the full genome of the FPV genome from two of these cats (No 10 and 11) showed an identical amino acid sequence of NS1, VP1 and VP2 to that of KP769859 strain (data not shown). The NS1 substitution was not found in intestinal FPV strains infecting three cats with a negative cerebral immunostaining (cats number 14, 18 and 23; Table 2). It has to be noted here that these three cats had a positive FPV PCR in brain tissues, but with high C_T_ values, especially when compared to C_T_ values in the ileum.

**Fig. 6:**
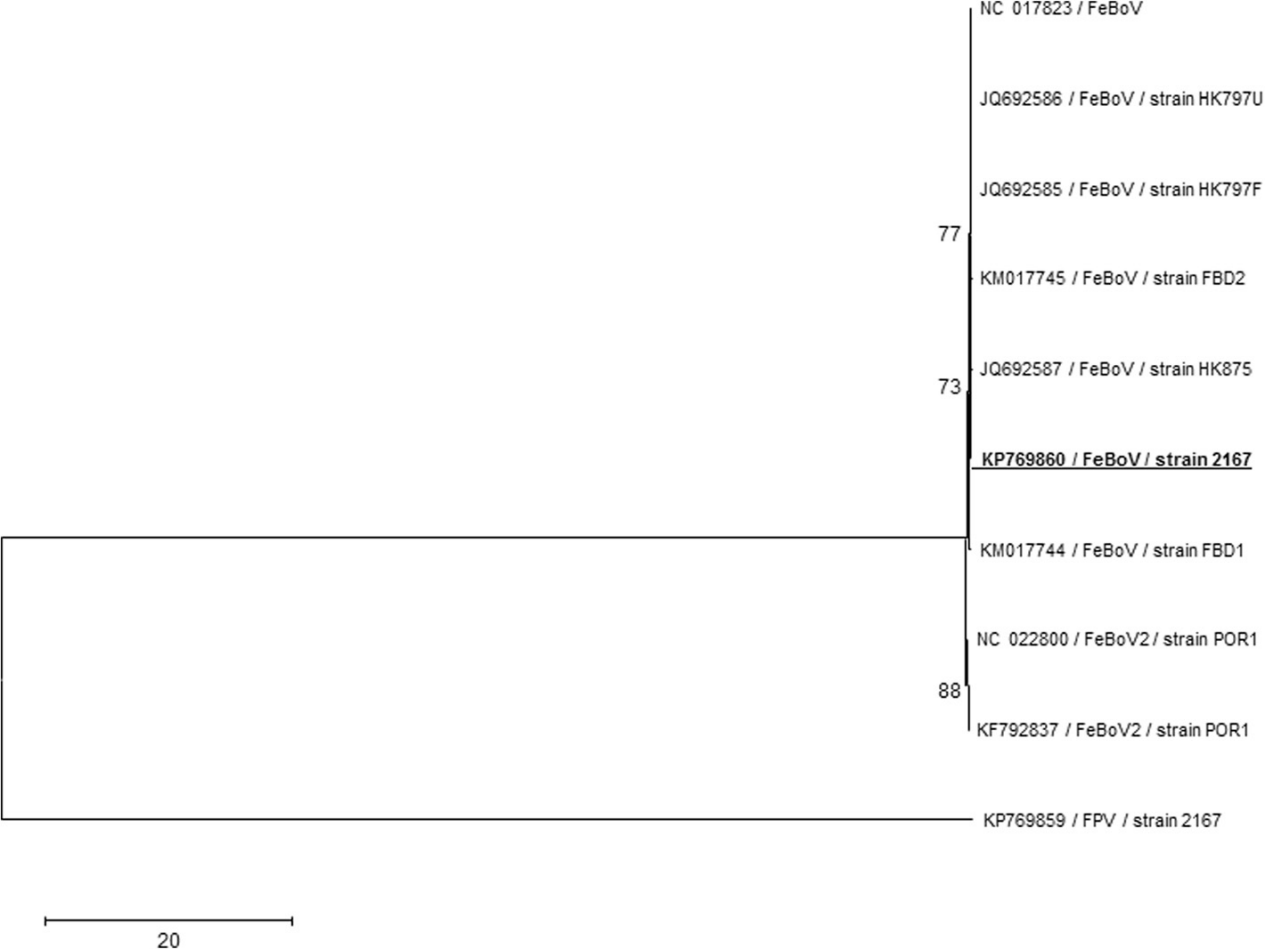
Maximum Likelihood phylogenetic analysis of feline bocavirus strain KP769860 based on partial genomic sequence. Currently available type 1 and type 2 feline bocavirus strains are included. A partial genome (4,001 nucleotides) was used for analysis. Statistical support of 1000 parallel Maximum Likelihood bootstrap replicates (≥70 %) is indicated at the nodes. FPV strain 2167 (Genbank accession number KP769859, also described in this study) is used as outgroup. Taxon information includes GenBank accession number, name/antigenic variant and strain. The feline bocavirus strain generated in this study is bolded and underlined. The scale bar represents nucleotide substitutions per site

Further analysis of the sequencing data from the cat brain infected by KP769859 FPV showed the presence of feline bocavirus. Subsequent PCR amplification with specific primers allowed the sequencing of around 80 % of the full genome (Genbank accession number: KP769860). Phylogenetic analysis based on the available partial genome allowed classification of this strain as a type 1 feline bocavirus (Fig. 6).

## Discussion

Feline panleukopenia virus has long been known to cause cerebellar hypoplasia in neonatal kittens through in utero or perinatal infection of the external germinal epithelium of the cerebellum [6, 9, 10]. Parvoviruses typically target highly mitotic cells such as those from intestinal crypts, bone marrow and lymphoid tissues [10]. This requirement explains why cerebellar hypoplasia is only observed in kittens infected perinatally or in utero and not in older cats. Cell cycle re-entry might be involved in the productive infection of Purkinje cells in kittens [11].

Extra-cerebellar lesions of the central nervous system associated with parvoviral infections were described in cats with demyelination of the spinal cord [12] and in cats with canine parvovirus replication in cerebral neurons [8, 13]. Such productive infections of cat cerebral neurons were strikingly associated with old CPV-2 strains, which seem to infect cats while they do not circulate in dog populations anymore, and have never been identified with CPV-2a and -2b variants, nor with FPV strains [8].

In the present study, we showed the presence of FPV proteins, likely VP1/VP2, given the antigens targeted by the CPV1-2A1 antibody used [7], in glial cells and neurons from diencephalic region of four cats, mostly the interthalamic adhesion. Similarly to observations reported with CPV-2 strains [8], the infection of cerebral neurons occurred in the absence of visible cerebellar infection or lesion. This is of utmost interest since some of the cats had a history of neurological signs before death. Moreover, one affected cat was adult, this last point raising questions about the possibility of a virus-induced re-entry of post-mitotic neurons into cell cycle [11]. To specifically address this point, we assessed the p27^Kip1^ expression of FPV-infected neurons by IHC. The cyclin-dependent kinase inhibitor p27^Kip1^ is expressed in cells that have exited the mitotic cycle [14]. Anti-p27^Kip1^ and anti-FPV immunostainings were realized on serial sections from the same samples and revealed that while most neurons were p27-positive in the nucleus, those infected by FPV were p27-negative, meaning a possible re-entry into the mitotic cycle of some neurons including those expressing FPV proteins [14]. The ability of the anti-human p27^Kip1^ antibody used in this study to efficiently and specifically bind the feline p27^Kip1^ protein had been demonstrated previously [11]. Further, relatively high concentrations of the antibody with some background reaction were necessary to get a significant nuclear staining. Recent evidence suggest that cell cycle re-entry in post-mitotic neurons may occur under specific circumstances [15]. Although the number of cases included in the present study is too low to be conclusive, this observation deserves to be further investigated. Besides, whether the lack of p27 expression is a cause or a consequence of the infection of these neurons by FPV remains to be determined.

Sequences analyses revealed that a unique L → S substitution was present in the NS1 protein from brain-infecting strains and not in other strains presented in this study. This substitution has thus far never been identified, neither in FPV nor in CPV strains. NS1 protein of human B19 parvovirus is known to cause cell cycle arrest at late S phase, which favors viral DNA replication [16] and is pro-apoptotic [17]. Similarly, Minute virus of Mice NS1 was associated with cell cycle arrest and p53 activation [18] and CPV-2 NS1 was shown to cause caspase-3 activation [19]. It is tempting to hypothesize that FPV NS1 could also be able to manipulate the cell cycle. The putative cause to effect of the NS1 mutation described for FPV strains in association with productive infection of cerebral neurons in this study deserves additional research. The potential link between this NS1 mutation and the lack of p27^Kip1^ expression in infected neurons should also be specifically addressed.

Lastly, the next-generation sequencing analysis of one parvovirus-infected cat’s brain tissue showed the co-infection by type 1 feline bocavirus. Bocaviruses are enteric viruses of the *Parvoviridae* family which have been described in several species and only recently in cats [20]. The pathogenic potential of these viruses remains to be determined [20]. Even if not confirmed by in situ histological techniques, the present study is the first report of nervous system infection by a bocavirus. The potential effects of the co-infection with FPV in the affected cat remains to be assessed.

## Conclusions

Overall, the results presented in this study show that, like CPV-2, FPV is able to infect cerebral neurons of young or adult cats without involvement of the cerebellum. The identification of a unique substitution in NS1 protein might be related to this unusual tropism but this hypothesis would require further investigations. This study also provides the first evidence of nervous tissue infection by a bocavirus.

## Methods

### Sample collection and real time PCR

During the year 2013, a total of 28 cats referred for necropsy to the Veterinary Pathology Department of the University of Liège, Belgium, were confirmed as cases of feline panleukopenia. Samples of brain, cerebellum, spleen, small intestine, mesenteric lymph node, liver, kidney, lung and myocardium were collected and stored at −80 °C for subsequent PCR analysis or fixed in 10 % formalin, routinely processed and embedded in paraffin for histopathological evaluation.

The feline panleukopenia virus genome was detected using a Taqman real time PCR assay previously described [21]. In the Taqman probe, BHQ1 was used as a quencher.

All studies were in accordance with the guidelines of the Institutional Animal Care and Use Committees of the University of Liège.

### Immunohistochemical staining

The organs examined for histopathology were subsequently submitted to immunohistochemical analysis. A commercial mouse monoclonal anti-parvovirus antibody (clone CPV1-2A1, sc57961, Santa Cruz, Dallas, Texas, USA) [7] and a polyclonal rabbit anti-human p27^Kip1^ antibody (ab7961, Abcam, Cambridge, United Kingdom) [11] were used as primary antibodies (dilution 1:50, for both). Goat anti-mouse or goat anti-rabbit serum (dilution 1/160, 323-005-024 and 323-005-024, respectively, Jackson ImmunoResearch, West Grove, USA) was used as secondary antibody. Binding was visualized using the peroxidase anti-peroxidase method with diaminobenzidine as the chromogen; sections were counterstained with Mayer hematoxylin. Cerebellar sections from an FPV-infected kitten with cerebellar hypoplasia were used as a positive control for the anti-parvovirus immunohistochemistry [7, 11], and sections from a fetal cerebellum (estimated gestational age, 54 days) were used as a positive control for the anti-p27^Kip1^ immunohistochemistry [11].

### Next-generation sequencing, confirmation PCRs and sequence analysis

Brain samples from three cats (5, 10, 11) for which sufficient amounts of tissue were available were submitted to next-generation sequencing. Briefly, 500 mg of tissue were homogenized in 1 ml of 1x DNase buffer (Life Technologies, Ghent, Belgium) for 5 min at 30Hz using a TissueLyser II device (Qiagen, Hilden, Germany). After centrifugation for 10 min at 11,000 g, supernatant was collected and filtered using a 0.2 μm filter (Pall Corporation, Newquay, United Kingdom). Viral particles were concentrated using a Microcon-100 kDa column (Merck Millipore, Billerica, USA) for 30 min at 500 g. Viral particles were washed once with 100 μl of 1x DNase buffer for 20 min at 500 g and eluted by inverting the column for 3 min at 1000 g. Turbo™Dnase (Life Technologies, Ghent, Belgium) and RNase A/T1 (Thermo Scientific, Waltham, USA) were added to the elution at a 1:50 dilution. The mixture was stored for 1 h at 37 °C. Viral particles were then digested using proteinase K and total DNA was extracted using NucleoSpin Tissue kit (Macherey-Nagel, Düren, Germany) according to manufacturer’s instructions. Libraries were prepared using Ion Plus Fragment Library Kit and sequencing was performed with the Ion Torrent PGM technology (Life Technologies, Ghent, Belgium). Full genome assembly and sequence analysis were performed with Geneious 8.0.5 (Biomatters, Auckland, New Zealand). Phylogenetic analyses were realized using MEGA 6.0 [22]. A nucleotide sequence identity matrix was produced with Sequence Demarcation Tool version 1.2 [23].

A confirmation PCR was used to verify a point mutation in NS1 coding sequence. Primers (Table 3) were designed using Primer3 [24]. PCRs were performed using HotStarTaq Plus Master Mix (Qiagen, Hilden, Germany) and conditions were as follows: a denaturation step at 95 °C for 5 min, followed by 45 cycles of 95 °C for 20 s, 55 °C for 45 s, 72 °C for 2 min, and final elongation 5 min at 72 °C. Sanger sequencing of the amplicons was performed by GATC Biotech (Konstanz, Germany).

**Table 3:** Primers used for amplification and sequencing of feline bocavirus (design/numbering based on GenBank accession number KM017745) and for confirmation of the substitution in NS1 coding sequence of FPV (design/numbering based on GenBank accession number KP769859)

Forward Primer	Sequence	Position	Reverse primer	Sequence	Position
BocaF1	ACAATGCCTGGACCTGAATC	481 → 500	BocaR1	TTTTACTCATTCTGGCATTCACA	1,276 → 1,254
BocaF2	CTAGTCAGGGACGAACCAA	1,141 → 1,159	BocaR2	CCATTGAGTATGAAAGCCAACT	2,103 → 2,082
BocaF3	CACGTCTAGAGAGCACCTTTGG	1,994 → 2,015	BocaR3	ATCAACCTCCATGGCAACC	3,118 → 3,100
BocaF4	DGGCATTTTAGCATGGGCGAA	2,966 → 2,986	BocaR4	GAAAATACCCGTATTGCGGAAGT	4,113 → 4,091
BocaF5	TACAGCGGGTGTACACATTT	3,9864,005	BocaR5	GTAGCAGTGTGGAGGGTGT	5,368 → 5,350
ParvF3	CAACCAATAAGAGACAGAATGTTGA	1,785 → 1,809	ParvR3	CCCCCACTTTACTAACACACC	2,342 → 2,322

Besides, primers were designed (Table 3) to amplify the genome of a feline bocavirus identified in the brain of one cat by next-generation sequencing. PCR/sequencing conditions were as described for panleukopenia virus.

### Ethical consideration

According to the law of the Walloon region in Belgium [25], standard diagnostic procedures performed on dead animals or animal tissues do not require permission from the Ethical Board. Cats included in this study were specifically referred by field veterinarians to the Faculty, in agreement with the owners, for pathological evaluation (necropsy and histopathology) and diagnosis of feline panleukopenia.
